# Anti NMDA-receptor encephalitis: a severe case (34 year-old male) with psychotic disorders

**DOI:** 10.1186/1471-2334-14-S7-P89

**Published:** 2014-10-15

**Authors:** Cleo Roşculeț, Andrei Rogoz, Ana Maria Petrescu, Cătălin Apostolescu, Marius Radu, Raluca Zlotea, Doina Iovănescu, Cristina Panea

**Affiliations:** 1 National Institute for Infectious Diseases "Prof. Dr. Matei Balş", Bucharest, Romania; 2 Elias University Emergency Hospital, Bucharest, Romania

## Background

Acute encephalitis associated with antibodies to the N-methyl-D-aspartate subtype of glutamate receptor is a recently described condition, the majority of cases presenting as a paraneoplastic syndrome in young females.

## Case report

We report the case of a 34 years-old male with anti-NMDA receptor encephalitis who exhibited the classical pattern. The patient described psychiatric symptoms, seizures, movement disorders, altered state of consciousness and autonomic dysfunction, over the course of 40-50 days. After a 20-day hospitalization in a psychiatric ward, the case was redirected to our infectious disease – intensive care facility, where he required advanced life support. The interdisciplinary team of infectious diseases, intensive care and neurology specialists raised the diagnosis suspicion of encephalitis. The patient received empirical corticotherapy and therapeutic plasma exchange, with significant clinical improvement in the second week of treatment, coinciding with the diagnostic confirmation by positive results for anti-NMDA receptor antibodies in both serum and cerebrospinal fluid.

The patient continued his recovery in a specialized neurology department. Infectious disease specialists are often confronted with encephalitis of unknown etiology.

## Conclusion

Collaboration with neurologists and psychiatrists with knowledge of this disorder is very important for an early diagnosis and treatment, as full recovery is a possible outcome for these patients with the correct case management.

## Consent

Written informed consent was obtained from the patient for publication of this Case report and any accompanying images. A copy of the written consent is available for review by the Editor of this journal.

